# Eth­oxy­carbonyl­methyl 3-(4-chloro­benzyl­idene)dithio­carbazate

**DOI:** 10.1107/S160053681003549X

**Published:** 2010-09-11

**Authors:** Masoumeh Tabatabaee, Mahboubeh A. Sharif, Robabeh Khalili, Masood Parvez

**Affiliations:** a Scientific Society of Nanotechnology, Islamic Azad University, Yazd Branch, Yazd, Iran; b Department of Chemistry, Islamic Azad University, Qom Branch, Qom, Iran; c Department of Chemistry, Islamic Azad University, Yazd Branch, Yazd, Iran; d Department of Chemistry, The University of Calgary, 2500 University Drive NW, Calgary, Alberta, Canada T2N 1N4

## Abstract

Mol­ecules of the title compound, C_12_H_13_ClN_2_O_2_S_2_, are linked into centrosymmetric dimers by pairs of inter­molecular N—H⋯S hydrogen bonds. In the crystal structure, there are π–π stacking inter­actions between symmetry-related benzene rings with a centroid–centroid distance of 3.7305 (13) Å, a perpendicular distance between the planes of 3.2851 (9) Å and a slippage of 1.768 Å. The structure is further stabilized by weak inter­molecular C—H⋯O hydrogen bonds.

## Related literature

For the biological activity of related compounds, see: Gülerman *et al.* (2001 ▶); Duran *et al.* (2002 ▶). For related structures, see: Tabatabaee *et al.* (2006 ▶, 2007 ▶, 2008 ▶, 2009 ▶).
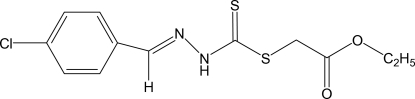


## Experimental

### 

#### Crystal data

C_12_H_13_ClN_2_O_2_S_2_
*M* *_r_* = 316.81
Triclinic, 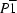

*a* = 7.3425 (3) Å
*b* = 10.3894 (4) Å
*c* = 10.6457 (5) Å
α = 116.535 (2)°
β = 95.049 (2)°
γ = 94.955 (2)°
*V* = 716.50 (5) Å^3^
*Z* = 2
Mo *K*α radiation
μ = 0.56 mm^−1^
*T* = 173 K
0.12 × 0.08 × 0.06 mm

#### Data collection

Nonius KappaCCD diffractometer with APEXII CCD
Absorption correction: multi-scan (*SORTAV*; Blessing, 1997 ▶) *T*_min_ = 0.936, *T*_max_ = 0.967
12341 measured reflections
3996 independent reflections
3361 reflections with *I* > 2σ(*I*)
*R*_int_ = 0.031

#### Refinement

*R*[*F* ^2^ > 2σ(*F* ^2^)] = 0.045
*wR*(*F* ^2^) = 0.104
*S* = 1.09
3996 reflections
173 parameters
H-atom parameters constrained
Δρ_max_ = 0.47 e Å^−3^
Δρ_min_ = −0.26 e Å^−3^

### 

Data collection: *COLLECT* (Hooft, 1998 ▶); cell refinement: *DENZO* (Otwinowski & Minor, 1997 ▶); data reduction: *SCALEPACK* (Otwinowski & Minor, 1997 ▶); program(s) used to solve structure: *SIR92* (Altomare *et al.*, 1994 ▶); program(s) used to refine structure: *SHELXL97* (Sheldrick, 2008 ▶); molecular graphics: *ORTEP-3* (Farrugia, 1997 ▶); software used to prepare material for publication: *SHELXL97*.

## Supplementary Material

Crystal structure: contains datablocks global, I. DOI: 10.1107/S160053681003549X/lh5124sup1.cif

Structure factors: contains datablocks I. DOI: 10.1107/S160053681003549X/lh5124Isup2.hkl

Additional supplementary materials:  crystallographic information; 3D view; checkCIF report

## Figures and Tables

**Table 1:** Hydrogen-bond geometry (Å, °)

*D*—H⋯*A*	*D*—H	H⋯*A*	*D*⋯*A*	*D*—H⋯*A*
N2—H2*N*⋯S1^i^	0.88	2.52	3.3669 (17)	161
C3—H3⋯O1^ii^	0.95	2.48	3.410 (2)	166
C9—H9*B*⋯O1^iii^	0.99	2.55	3.153 (3)	119

Symmetry codes: (i) 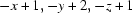; (ii) 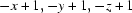; (iii) 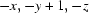.

**Fig. 1.**
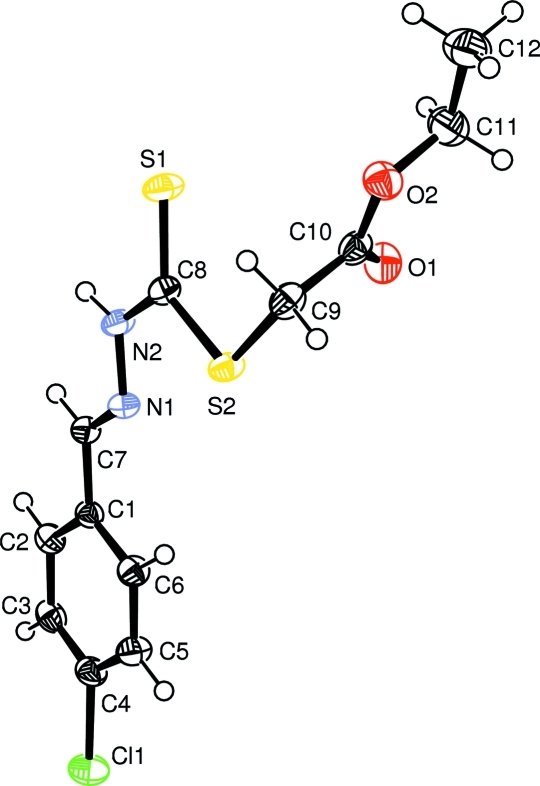
ORTEP-3 (Farrugia, 1997) drawing of the title compound with displacement ellipsoids plotted at 50% probability level.

**Fig. 2.**
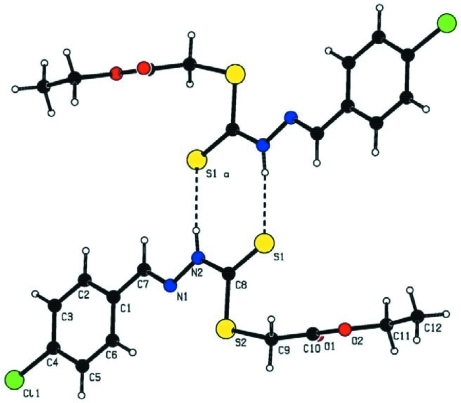
Dimeric arrangement of the title compound formed by intermolecular N—H···S hydrogen bonds and shown as dashed lines. S1a is related by the symmetry code (1-x, 2-y, 1-z).

**Fig. 3.**
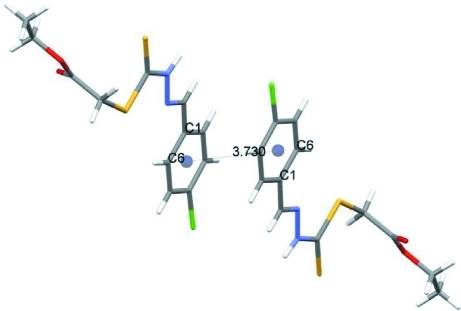
Representation of π-π stacking interactions in the crystal structure of the title compound

**Fig. 4.**
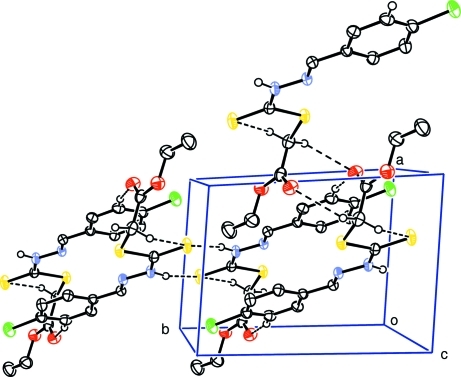
Unit cell packing of the title compound showing hydrogen bonding interactions as dashed lines.

## supplementary crystallographic information

### Comment

Thiones of nitrogen-containing heterocycles have attracted the attention of researchers in recent years because of their synthetic possibilities and useful properties. Several compounds containing sulfur and nitrogen atoms are anti-inflammatory, sedative, antibacterial, antiviral, or antitumor and synthesis of the corresponding iminic compounds could be of interest from the viewpoint of chemical reactivity and biological activity (Gülerman *et al.*, 2001; Duran *et al.*, 2002). In a sequence of studies, we have investigated the synthesis and crystal structures of several Schiff bases derived from 4-amino-5-methyl-2*H*-1,2,4-triazole-3(4*H*)-thione (AMTT) and 4-amino-6-methyl-3-thio-3,4-dihydro-1,2,4-triazin-5(2*H*)-one (AMTTO) (Tabatabaee *et al.*, 2006; 2007; 2008; 2009). Here, we report our results for the synthesis and crystal structure of a new iminic compounds derived from *N*-aminorhodanine, (I).

In the title molecule (Fig. 1) bond distances and angles are unexceptional and agree with the corresponding bond distances and angles reported in the related compounds (Tabatabaee *et al.*, 2006; 2007; 2008; 2009). In the solid state, intermolecular N—H···S hydrogen bonds in the title compound link the molecules lying about inversion centers leading to centrosymmetric dimers (Tab. 1 & Fig. 2). Moreover, the benzene rings C1–C6 and C1^i^–C6^i^ (^i^= 2 - *x*, 1 - *y*, 1 - *z*) show π-π stacking interactions (Fig. 3) with centroid-centroid distance 3.7305 (13) Å, the angle between the planes 0 °; the perpendicular distance between the planes 3.2851 (9) Å and the slippage 1.768 Å. The structure is further stabilized by intermolecular hydrogen bonding of C—H···O type (Tab. 1); unit cell packing showing hydrogen bonding interactions has been presented in Figure 4.

### Experimental

A solution of *N*-aminorhodanine (5 mmol) in EtOH (20 ml) was treated with 2-chlorobenzaldehyde (5 mmol) and the resulting mixture was acidified with 37% hydrochloric acid (0.2 ml). The reaction mixture was refluxed for 8 h. After completion of the reaction, the solid residue was filtered, washed with cold ethanol (10 ml) and recrystallized from EtOH.

### Refinement

The H-atoms were visible in difference Fourier maps but were included in the refinement in geometrically idealized positions with distances N—H = 0.88 Å and C—H = 0.95, 0.98 and 0.99 Å for aryl, methyl and methylene type H-atoms, respectively. The H-atoms were assigned *U*_iso_ = 1.2 × *U*_eq_ of the parent atoms. The final difference map was free of chemically significant features.

### Crystal data

**Table:**

C_12_H_13_ClN_2_O_2_S_2_	*Z* = 2
*M**_r_* = 316.81	*F*(000) = 328
Triclinic, *P*1	*D*_x_ = 1.468 Mg m^−^^3^
Hall symbol: -P 1	Mo *K*α radiation, λ = 0.71073 Å
*a* = 7.3425 (3) Å	Cell parameters from 3737 reflections
*b* = 10.3894 (4) Å	θ = 1.0–29.6°
*c* = 10.6457 (5) Å	µ = 0.56 mm^−^^1^
α = 116.535 (2)°	*T* = 173 K
β = 95.049 (2)°	Block, colorless
γ = 94.955 (2)°	0.12 × 0.08 × 0.06 mm
*V* = 716.50 (5) Å^3^	

### Data collection

**Table:**

Nonius KappaCCD diffractometer with APEXII CCD	3996 independent reflections
Radiation source: fine-focus sealed tube	3361 reflections with *I* > 2σ(*I*)
graphite	*R*_int_ = 0.031
ω and φ scans	θ_max_ = 29.7°, θ_min_ = 2.2°
Absorption correction: multi-scan (*SORTAV*; Blessing, 1997)	*h* = −10→10
*T*_min_ = 0.936, *T*_max_ = 0.967	*k* = −14→14
12341 measured reflections	*l* = −14→14

### Refinement

**Table:**

Refinement on *F*^2^	Primary atom site location: structure-invariant direct methods
Least-squares matrix: full	Secondary atom site location: difference Fourier map
*R*[*F*^2^ > 2σ(*F*^2^)] = 0.045	Hydrogen site location: difference Fourier map
*wR*(*F*^2^) = 0.104	H-atom parameters constrained
*S* = 1.09	*w* = 1/[σ^2^(*F*_o_^2^) + (0.0252*P*)^2^ + 0.6628*P*] where *P* = (*F*_o_^2^ + 2*F*_c_^2^)/3
3996 reflections	(Δ/σ)_max_ < 0.001
173 parameters	Δρ_max_ = 0.47 e Å^−^^3^
0 restraints	Δρ_min_ = −0.26 e Å^−^^3^

### Special details

**Table:**

Geometry. All e.s.d.'s (except the e.s.d. in the dihedral angle between two l.s. planes) are estimated using the full covariance matrix. The cell e.s.d.'s are taken into account individually in the estimation of e.s.d.'s in distances, angles and torsion angles; correlations between e.s.d.'s in cell parameters are only used when they are defined by crystal symmetry. An approximate (isotropic) treatment of cell e.s.d.'s is used for estimating e.s.d.'s involving l.s. planes.
Refinement. Refinement of *F*^2^ against ALL reflections. The weighted *R*-factor *wR* and goodness of fit *S* are based on *F*^2^, conventional *R*-factors *R* are based on *F*, with *F* set to zero for negative *F*^2^. The threshold expression of *F*^2^ > σ(*F*^2^) is used only for calculating *R*-factors(gt) *etc*. and is not relevant to the choice of reflections for refinement. *R*-factors based on *F*^2^ are statistically about twice as large as those based on *F*, and *R*- factors based on ALL data will be even larger.

### Fractional atomic coordinates and isotropic or equivalent isotropic displacement parameters (Å^2^)

**Table:**

	*x*	*y*	*z*	*U*_iso_*/*U*_eq_	
Cl1	0.89852 (9)	0.10405 (6)	0.44205 (7)	0.04491 (16)	
S1	0.37647 (8)	0.94428 (5)	0.28183 (6)	0.03285 (14)	
S2	0.37807 (7)	0.61903 (5)	0.12742 (5)	0.02750 (12)	
O1	−0.0267 (2)	0.65882 (18)	0.13349 (16)	0.0355 (3)	
O2	−0.0309 (2)	0.76565 (17)	−0.00860 (17)	0.0348 (3)	
N1	0.5839 (2)	0.65588 (17)	0.36811 (18)	0.0251 (3)	
N2	0.5413 (2)	0.78764 (17)	0.38235 (17)	0.0259 (3)	
H2N	0.5813	0.8681	0.4610	0.031*	
C1	0.7182 (2)	0.5188 (2)	0.4706 (2)	0.0224 (3)	
C2	0.8110 (3)	0.5168 (2)	0.5894 (2)	0.0260 (4)	
H2	0.8383	0.6039	0.6763	0.031*	
C3	0.8640 (3)	0.3890 (2)	0.5824 (2)	0.0280 (4)	
H3	0.9271	0.3880	0.6637	0.034*	
C4	0.8236 (3)	0.2634 (2)	0.4555 (2)	0.0292 (4)	
C5	0.7266 (3)	0.2612 (2)	0.3364 (2)	0.0306 (4)	
H5	0.6973	0.1733	0.2505	0.037*	
C6	0.6734 (3)	0.3889 (2)	0.3448 (2)	0.0263 (4)	
H6	0.6057	0.3884	0.2642	0.032*	
C7	0.6691 (3)	0.6550 (2)	0.4777 (2)	0.0244 (4)	
H7	0.7005	0.7428	0.5636	0.029*	
C8	0.4379 (3)	0.7910 (2)	0.2744 (2)	0.0238 (4)	
C9	0.2345 (3)	0.6622 (2)	0.0105 (2)	0.0291 (4)	
H9A	0.2984	0.7477	0.0061	0.035*	
H9B	0.2172	0.5795	−0.0861	0.035*	
C10	0.0471 (3)	0.6945 (2)	0.0554 (2)	0.0281 (4)	
C11	−0.2133 (3)	0.8016 (3)	0.0258 (3)	0.0419 (5)	
H11A	−0.2092	0.8571	0.1298	0.050*	
H11B	−0.3024	0.7118	−0.0090	0.050*	
C12	−0.2709 (4)	0.8910 (3)	−0.0444 (3)	0.0506 (6)	
H12A	−0.3951	0.9147	−0.0251	0.061*	
H12B	−0.2716	0.8359	−0.1470	0.061*	
H12C	−0.1839	0.9809	−0.0071	0.061*	

### Atomic displacement parameters (Å^2^)

**Table:**

	*U*^11^	*U*^22^	*U*^33^	*U*^12^	*U*^13^	*U*^23^
Cl1	0.0472 (3)	0.0344 (3)	0.0633 (4)	0.0143 (2)	0.0028 (3)	0.0306 (3)
S1	0.0483 (3)	0.0196 (2)	0.0283 (3)	0.0074 (2)	−0.0043 (2)	0.01017 (19)
S2	0.0359 (3)	0.0192 (2)	0.0239 (2)	0.00528 (18)	0.00303 (19)	0.00682 (18)
O1	0.0363 (8)	0.0418 (9)	0.0338 (8)	0.0012 (7)	0.0033 (6)	0.0230 (7)
O2	0.0362 (8)	0.0390 (8)	0.0361 (8)	0.0066 (7)	0.0033 (6)	0.0233 (7)
N1	0.0275 (8)	0.0193 (7)	0.0301 (8)	0.0055 (6)	0.0036 (6)	0.0124 (7)
N2	0.0331 (9)	0.0173 (7)	0.0253 (8)	0.0037 (6)	0.0000 (6)	0.0087 (6)
C1	0.0212 (8)	0.0232 (8)	0.0266 (9)	0.0048 (7)	0.0044 (7)	0.0143 (7)
C2	0.0254 (9)	0.0293 (9)	0.0253 (9)	0.0058 (7)	0.0051 (7)	0.0136 (8)
C3	0.0243 (9)	0.0367 (10)	0.0311 (10)	0.0070 (8)	0.0045 (7)	0.0219 (9)
C4	0.0263 (9)	0.0290 (10)	0.0405 (11)	0.0073 (8)	0.0071 (8)	0.0223 (9)
C5	0.0365 (11)	0.0243 (9)	0.0306 (10)	0.0066 (8)	0.0039 (8)	0.0121 (8)
C6	0.0293 (9)	0.0270 (9)	0.0251 (9)	0.0046 (7)	0.0013 (7)	0.0144 (8)
C7	0.0238 (8)	0.0215 (8)	0.0268 (9)	0.0047 (7)	0.0048 (7)	0.0098 (7)
C8	0.0274 (9)	0.0207 (8)	0.0239 (9)	0.0030 (7)	0.0045 (7)	0.0108 (7)
C9	0.0361 (10)	0.0271 (9)	0.0215 (9)	0.0003 (8)	0.0013 (8)	0.0098 (8)
C10	0.0336 (10)	0.0238 (9)	0.0237 (9)	−0.0009 (8)	−0.0020 (8)	0.0099 (8)
C11	0.0372 (12)	0.0431 (13)	0.0504 (14)	0.0093 (10)	0.0063 (10)	0.0252 (12)
C12	0.0539 (16)	0.0440 (14)	0.0556 (16)	0.0164 (12)	0.0016 (13)	0.0237 (13)

### Geometric parameters (Å, °)

**Table:**

Cl1—C4	1.739 (2)	C3—C4	1.380 (3)
S1—C8	1.6618 (19)	C3—H3	0.9500
S2—C8	1.7548 (19)	C4—C5	1.389 (3)
S2—C9	1.792 (2)	C5—C6	1.382 (3)
O1—C10	1.201 (2)	C5—H5	0.9500
O2—C10	1.339 (2)	C6—H6	0.9500
O2—C11	1.455 (3)	C7—H7	0.9500
N1—C7	1.279 (2)	C9—C10	1.513 (3)
N1—N2	1.375 (2)	C9—H9A	0.9900
N2—C8	1.337 (2)	C9—H9B	0.9900
N2—H2N	0.8800	C11—C12	1.493 (3)
C1—C2	1.392 (2)	C11—H11A	0.9900
C1—C6	1.398 (3)	C11—H11B	0.9900
C1—C7	1.462 (2)	C12—H12A	0.9800
C2—C3	1.388 (3)	C12—H12B	0.9800
C2—H2	0.9500	C12—H12C	0.9800
			
C8—S2—C9	100.82 (9)	C1—C7—H7	120.1
C10—O2—C11	115.16 (17)	N2—C8—S1	122.37 (14)
C7—N1—N2	116.85 (16)	N2—C8—S2	112.84 (13)
C8—N2—N1	118.65 (16)	S1—C8—S2	124.78 (11)
C8—N2—H2N	120.7	C10—C9—S2	113.36 (14)
N1—N2—H2N	120.7	C10—C9—H9A	108.9
C2—C1—C6	119.06 (17)	S2—C9—H9A	108.9
C2—C1—C7	120.29 (17)	C10—C9—H9B	108.9
C6—C1—C7	120.66 (16)	S2—C9—H9B	108.9
C3—C2—C1	120.73 (18)	H9A—C9—H9B	107.7
C3—C2—H2	119.6	O1—C10—O2	123.7 (2)
C1—C2—H2	119.6	O1—C10—C9	126.00 (19)
C4—C3—C2	118.96 (18)	O2—C10—C9	110.25 (17)
C4—C3—H3	120.5	O2—C11—C12	107.7 (2)
C2—C3—H3	120.5	O2—C11—H11A	110.2
C3—C4—C5	121.55 (18)	C12—C11—H11A	110.2
C3—C4—Cl1	119.71 (15)	O2—C11—H11B	110.2
C5—C4—Cl1	118.73 (16)	C12—C11—H11B	110.2
C6—C5—C4	119.01 (19)	H11A—C11—H11B	108.5
C6—C5—H5	120.5	C11—C12—H12A	109.5
C4—C5—H5	120.5	C11—C12—H12B	109.5
C5—C6—C1	120.63 (17)	H12A—C12—H12B	109.5
C5—C6—H6	119.7	C11—C12—H12C	109.5
C1—C6—H6	119.7	H12A—C12—H12C	109.5
N1—C7—C1	119.89 (17)	H12B—C12—H12C	109.5
N1—C7—H7	120.1		
			
C7—N1—N2—C8	−174.09 (18)	C2—C1—C7—N1	−179.95 (18)
C6—C1—C2—C3	2.1 (3)	C6—C1—C7—N1	0.2 (3)
C7—C1—C2—C3	−177.71 (18)	N1—N2—C8—S1	179.15 (14)
C1—C2—C3—C4	0.1 (3)	N1—N2—C8—S2	−1.3 (2)
C2—C3—C4—C5	−1.9 (3)	C9—S2—C8—N2	177.22 (15)
C2—C3—C4—Cl1	176.88 (15)	C9—S2—C8—S1	−3.25 (16)
C3—C4—C5—C6	1.5 (3)	C8—S2—C9—C10	−73.08 (15)
Cl1—C4—C5—C6	−177.31 (16)	C11—O2—C10—O1	1.6 (3)
C4—C5—C6—C1	0.8 (3)	C11—O2—C10—C9	179.48 (17)
C2—C1—C6—C5	−2.6 (3)	S2—C9—C10—O1	−20.3 (3)
C7—C1—C6—C5	177.28 (18)	S2—C9—C10—O2	161.95 (14)
N2—N1—C7—C1	179.74 (16)	C10—O2—C11—C12	175.16 (19)

### Hydrogen-bond geometry (Å, °)

**Table:**

*D*—H···*A*	*D*—H	H···*A*	*D*···*A*	*D*—H···*A*
N2—H2N···S1^i^	0.88	2.52	3.3669 (17)	161
C3—H3···O1^ii^	0.95	2.48	3.410 (2)	166
C9—H9B···O1^iii^	0.99	2.55	3.153 (3)	119
C9—H9A···S1	0.99	2.69	3.066 (2)	103

Symmetry codes: (i) −*x*+1, −*y*+2, −*z*+1; (ii) −*x*+1, −*y*+1, −*z*+1; (iii) −*x*, −*y*+1, −*z*.
